# Effects of temperature and drought on early life stages in three species of butterflies: Mortality of early life stages as a key determinant of vulnerability to climate change?

**DOI:** 10.1002/ece3.3588

**Published:** 2017-11-11

**Authors:** Michael Klockmann, Klaus Fischer

**Affiliations:** ^1^ Zoological Institute and Museum University of Greifswald Greifswald Germany

**Keywords:** copper butterfly, desiccation resistance, early developmental stages, environmental stress, food stress, heat stress, *Lycaena* species

## Abstract

Anthropogenic climate change poses substantial challenges to biodiversity conservation. Well‐documented responses include phenological and range shifts, and declines in cold but increases in warm‐adapted species. Thus, some species will suffer while others will benefit from ongoing change, although the biological features determining the prospects of a given species under climate change are largely unknown. By comparing three related butterfly species of different vulnerability to climate change, we show that stress tolerance during early development may be of key importance. The arguably most vulnerable species showed the strongest decline in egg hatching success under heat and desiccation stress, and similar pattern also for hatchling mortality. Research, especially on insects, is often focussed on the adult stage only. Thus, collating more data on stress tolerance in different life stages will be of crucial importance for enhancing our abilities to predict the fate of particular species and populations under ongoing climate change.

## 1 INTRODUCTION

The Earth's mean surface temperature and the frequency of extreme weather events such as heat waves have already increased as a result of anthropogenic climate change (Hansen, Sato, & Ruedy, 2012; McKechnie & Wolf, 2010; Meehl et al., 2007). These changes have in turn resulted in phenological and range shifts as well as abundance changes in a plethora of species (Chown et al., 2010; Parmesan & Yohe, 2003; Sunday, Bates, & Dulvy, 2012; Thomas, 2010). In this context, the extreme temperatures associated with heat waves seem to be more important than changes in mean temperatures because they typically exert a much stronger selection pressure (Anderson, Collinge, Hoffmann, Kellett, & McKechnie, 2003; Kellermann et al., 2012; Zimmermann et al., 2009). However, in addition to increasing temperature stress, terrestrial organisms will likely experience higher levels of desiccation and food stress, due to detrimental effects of drought periods on water supplies and food plant quality and availability (Clusella‐Trullas, Blackburn, & Chown, 2011; Hoffmann, Chown, & Clusella‐Trullas, 2013).

The changes outlined above are considered to be a major threat to biodiversity (Pimm et al., 2014; Thomas et al., 2004). However, while some species will suffer, others may benefit from ongoing climate change (e.g., many warm‐adapted species). Thus, responses to climate change are likely species specific, probably depending on a given species ability to cope with extreme temperatures, desiccation, and associated food stress (Anderson et al., 2003; Coumou & Rahmstorf, 2012). To identify which species are most at risk from climate change is of prime importance to predict the future consequences of ongoing climate change (Deutsch et al., 2008; Chown et al., 2010; Beaumont & Hughes 2002; Hoffmann & Sgrò 2011; Rosset & Oertli 2011). Unfortunately, the specific biological features determining whether a given species is becoming a “winner” or “loser” of climate change are largely unknown (Brook et al., 2009; Williams, Shoo, Isaac, Hoffmann, & Langham, 2008). In this context, it should be noted that research, especially on insects, is often focussed on the adult stage only, which may bias predictions regarding a species’ survival under climate change (Kingsolver, 2009; Kingsolver et al., 2011; Klockmann, Günter, & Fischer, 2017; Radchuk, Turlure, & Schtickzelle, 2013). Typically, stress tolerance varies throughout ontogeny in insects (Bowler & Terblanche, 2008; Kingsolver et al., 2011), such that it is necessary to identify the most vulnerable life stage (Bowler & Terblanche, 2008; Klockmann, Günter, et al., 2017; Radchuk et al., 2013). Here, early life stages, often facing high mortality, may be particularly crucial although temperature stress perceived early in life may not necessarily affect later life (Klockmann, Günter, et al., 2017; Potter, Davidowitz, & Arthur Woods, 2011). However, matters are complicated as other factors may also play an important role in the mortality of early life stages, for instance, the specific microclimatic conditions provided by the host plants (Potter, Davidowitz, & Woods, 2009; Smith, 1978; Woods, 2013).

**Figure 1:**
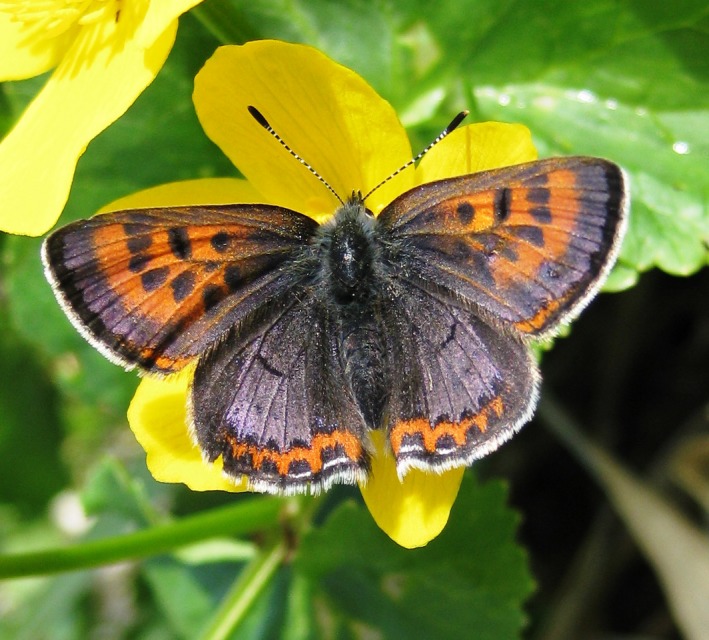
Photograph of a female *Lycaena helle* (the most threatened species of the three investigated ones) in the vicinity of Liebenscheid in western Germany (Photo by Klaus Fischer)

We here investigate the stress tolerance of early developmental stages in three species of Copper butterflies, namely *Lycaena tityrus* (Poda, 1761), *Lycaena dispar* (Haworth, 1802), and *Lycaena helle* (Denis & Schiffermüller, 1775; Figure 1). Currently, *L. tityrus* shows positive, *L. dispar* largely stable, and *L. helle* negative population trends (Brunzel, Bussmann, & Obergruber, 2008; Settele et al., 2008; Habel et al. 2011; Lindman et al., 2015). These differences seem to be associated with different distribution areas and habitat requirements, with *L. tityrus* inhabiting different types of habitat including hot and dry stands, *L. dispar* mainly wetlands, and *L. helle* cool and moist habitats (Ebert & Rennwald, 1991; Settele et al., 2008; Habel et al. 2011). Consequently, these species may also differ in (heat) stress tolerance and concomitantly in their vulnerability to climate change, ranging from low to high risk (Ebert & Rennwald, 1991; Settele et al., 2008; Habel et al. 2011; see further below and Table 1). We focus on early developmental stages because earlier studies showed that differences in vulnerability are unlikely to be caused by differential responses to thermal stress during larval and pupal development (Klockmann, Karajoli, Reimer, Kuczyk, & Fischer, 2016; Klockmann, Schröder, Karajoli, & Fischer, 2016) as well as adult stress resistance (Klockmann, Wallmeyer, & Fischer, 2017). We hypothesize that (i) mortality rates increase at higher temperature and additionally with reduced humidity in all species, (ii) and that *L. helle* will suffer most strongly from simulated heat and drought stress. Such species differences in the sensitivity to stress are statistically indicated by species × treatment interactions, for which we explicitly test here.

**Table 1:** Summary of key ecological and conservation attributes for *Lycaena tityrus*,* L. dispar*, and *L. helle*

	*L. tityrus*	*L. dispar*	*L. helle*
Geographical range	Eurasia	Eurasia	Eurasia
Range extent (northern latitude)	59–37°	62–40°	70–43°
Altitudinal preference	Indifferent	Lowlands	Mountainous
Principal habitat	Dry to moist grassland, heathland	Moist grassland, floodplains	Bogs, moist grassland
Temperature preference	Indifferent	±Thermophilic	Cold‐stenothermic
Humidity preference	Mesophilous	Hygrophilous	Hygrophilous
Larval host plant	*Rumex* spp.	*Rumex* spp.	*Bistorta officinalis*
Generations per year	1–3	1–2	1–2
Diapause stage	Larva	Larva	Pupa
Red list status (Germany)	Least concern	Vulnerable	Endangered
Current trend in Europe	Increasing	Largely stable	Decreasing
General vulnerability	Intermediate	±High	Very high
Vulnerability to climate change	Low risk	Intermediate risk	High risk

Note that all information on habitat preferences refers to the habitats in central Europe. Vulnerability refers to the general sensitivity to environmental including habitat change, while the last item explicitly draws on the expected impacts of climate change. The assessment of the vulnerability to climate change rests on the data summarized here (table adapted from Klockmann, Karajoli, et al., 2016).

## 2 MATERIALS AND METHODS

### 2.1 Study organisms and egg sampling

**Figure 2:**
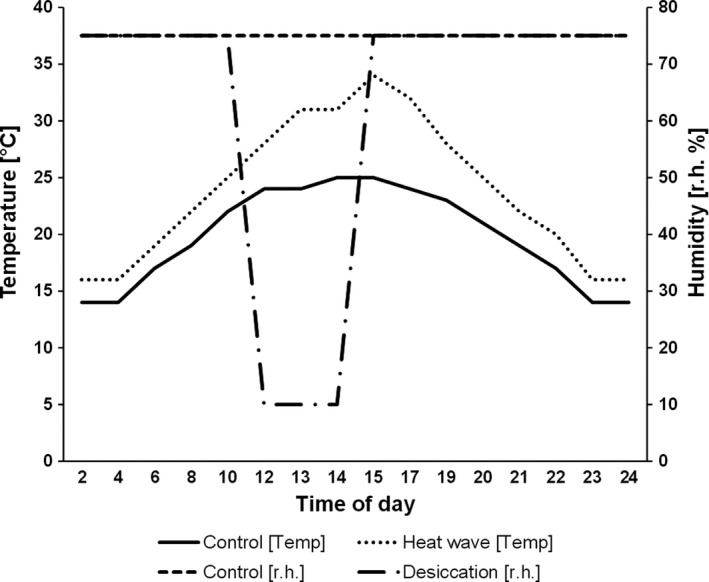
Graphical illustration of the conditions used to investigate stress tolerance of Copper butterfly eggs. Treatments lasted for 2 days and started 2 days after egg‐laying. For the desiccation treatment, control conditions were used except that the glass vials containing the eggs were transferred on two consecutive days to a box with silica gel to reduce the relative humidity

To investigate vulnerability to climate change, we used three species of Copper butterflies (*Lycaena* spp.; cf. Klockmann, Karajoli, et al., 2016). The Sooty Copper *L. tityrus* (Poda, 1761) is a widespread temperate‐zone butterfly, ranging from western Europe to central Asia (Ebert & Rennwald, 1991). The species has 1–3 generations per year and inhabits a variety of biotopes like grassland, sandy heathland, bogs, and open woodland (Brunzel et al., 2008; Settele et al., 2008). The principal larval host plant is *Rumex acetosa* L., but some congeneric plant species (e.g., *R. acetosella* L., *R. scutatus* L.) are utilized as well (Ebert & Rennwald, 1991; Settele et al., 2008; Tolman & Lewington, 2008). This species is not listed in the EU Habitat Directive and is considered least concern in the Red List of Germany (Settele et al., 2008; Binot‐Hafke et al., 2011; Table 1). *Lycaena tityrus* has recently colonized previously unoccupied mountain ranges in central Europe and is expanding its range northward in northeastern Europe (Brunzel et al., 2008; Settele et al., 2008). Because of these range expansions and its ability to inhabit even dry and hot habitats, the species is expected to benefit from climate change and its according vulnerability is consequently considered to be low. Mated females were caught in two bivoltine German populations in July 2014 in the vicinity of Ueckermünde (*N* = 10; N:53°44′; E:14°15′) and Greifswald (*N* = 12; N:54°2′; E:13°26′), and were transferred to Greifswald University for egg‐laying. Butterflies were kept in a climate cabinet (Sanyo MLR‐351H; Bad Nenndorf, Germany) under naturally fluctuating temperatures to improve ecological realism (i.e., control conditions: mean 19.4°C, 75% relative humidity, and L17:D7 photoperiod; Figure 2). For oviposition, females were placed individually in translucent 1‐L plastic pots and were provided with *R. acetosa* for egg‐laying and with fresh flowers (*Crepis* sp. L.*, Achillea millefolium* L.*, Bistorta officinalis* Delarbre*, Leucanthemum vulgare* LAM.), water, and a 20 vol% sucrose solution for adult feeding. Eggs were collected daily and transferred, separated by female, to small glass vials and kept under egg‐laying conditions until allocation to treatment groups.

The second species, the Large Copper *L. dispar* (Haworth, 1802), is a transpalaearctic butterfly, ranging from western Europe across temperate Asia to the Amur region and Korea (Ebert & Rennwald, 1991; Settele et al., 2008). The species has 1–2 generations per year (Lindman et al., 2015; Settele et al., 2008) and typically occurs in wetland habitats including lakeside and riverside areas (Lindman et al., 2015; Settele et al., 2008). Eggs are laid on nonacidic sorrels (e.g., *Rumex hydrolapathum* Huds., *R. crispus* L., *R. obtusifolius* L.; Lindman et al., 2015 and references therein). *Lycaena dispar* is listed in the EU Habitat Directive and is considered vulnerable in the Red List of Germany (Binot‐Hafke et al., 2011; Martin, Van Dyck, Dendoncker, & Titeux, 2013; Settele et al., 2008). Because of its association with wetland habitats and declines in some parts of its range, we assess the species’ vulnerability to climate change as being intermediate. Mated females were caught in two univoltine populations in July 2013 in Estonia, vicinity of Liispõllu (*N* = 9; N:58°15′; E:27°15′), and in Germany, vicinity of Anklam (*N* = 20; N:53°53′; E:13°42′), and were transferred to Greifswald University for egg‐laying. Butterflies were kept for egg‐laying as outlined above, using *R*. *hydrolapathum* as oviposition substrate.

The Violet Copper *L. helle* (Denis & Schiffermüller, 1775; Figure 1) is a boreal butterfly with 1–2 generations per year, ranging from central Europe, where it is a postglacial relict species, to Scandinavia and Northern Asia (Ebert & Rennwald, 1991; Settele et al., 2008). It is a hygrophilous butterfly colonizing mires, swampy grassland, and moist meadows (Ebert & Rennwald, 1991; Fischer, Beinlich, & Plachter, 1999; Settele et al., 2008). The only larval food plant in central Europe is *B. officinalis* (Fischer et al., 1999). The species is declining in large parts of its range (Van Swaay & Warren, 1999), is listed in the EU Habitat Directive, and is considered endangered in the Red List of Germany (Binot‐Hafke et al., 2011; Settele et al., 2008). Because of the strong population declines and its specialized habitat requirements, being confined to moist and cool stands, we consider the species’ vulnerability to climate change as being high (Habel et al. 2011). Mated females were caught in two populations in May 2014 in Belgium, vicinity of Baraque de Fraiture (*N* = 10; N:50°13′; E:14°15′), and in Germany, vicinity of Liebenscheid (*N* = 10; N:50°40′; E:8°04′), and were transferred to Greifswald University for egg‐laying. Butterflies were kept for egg‐laying as outlined above with the following exceptions. For oviposition, females were placed groupwise into translucent 20‐L plastic box and provided with *B. officinalis*. Keeping females individually resulted in very low egg numbers, which would have been insufficient for subsequent experiments.

### 2.2 Experimental design

We investigated egg and hatchling mortality with the eggs obtained using a split‐brood design for *L. tityrus* and *L. dispar*, while for *L. helle* eggs were randomly divided into groups. In the first experiment, we investigated the effects of heat and desiccation stress on egg mortality. Both stresses seem to be ecologically relevant in the egg stage. For testing, eggs were placed 2 days after laying into glass vials in groups of 10, using 9–20 replicates per treatment and population. Replicates consisted of random larvae in *L. helle* and full siblings in *Lycaena tityrus* and *L. dispar*. The three treatments used involved a (i) control, (ii) a heat (including an exposure for 2 days to a simulated heat wave), and a (iii) desiccation treatment, in which eggs were placed for 2 hr on two consecutive days into a box containing silica gel with a relative humidity of 10% to mimic a period of drought (Figure 2). The temperature cycles used are based on field data obtained in larval habitats of *L. helle* within the Westerwald mountain range in the years 2011 and 2013, where *L. tityrus* and *L. helle* naturally occur (Limberg & Fischer, 2014). Thus, we used a temperature cycle typical of average conditions in June (i.e., within larval period) as control and the data of a particularly hot June day to simulate a heat wave (Limberg & Fischer, 2014). Although the humidity chosen is very low, note the short exposure time mimicking conditions that may occur, for instance, during spells of direct sun exposure. Our field data obtained from larval habitats confirm that such low humidities are ecologically relevant. The absolute minimum relative humidity within larval habitats, based on 39 locations, was on average 6.1 ± 0.2% (Limberg & Fischer, 2014). Similar experimental setups have been repeatedly used before to assess desiccation resistance (Gomez, Sambucetti, Loeschcke, & Norry, 2015; Pichrtová, Kulichová, & Holzinger, 2014; Tejeda et al., 2016). Except from exposure to heat waves or low humidity, all eggs were kept under control conditions. Egg hatching success per glass vial (10 eggs) was scored under control conditions as percent.

In the second experiment, we investigated effects of heat and food stress on hatchling mortality. We used a food stress rather than a desiccation treatment here for ecological reasons. While eggs certainly suffer from low humidity, larvae may not as they are able to obtain water from plant material. We therefore decided to manipulate plant quality rather than humidity. Hatchlings were placed 2 days after hatching, separated by female for *L. tityrus* and *L. dispar*, in groups of 10 into translucent plastic boxes (250 ml) lined with moist tissue and containing a leaf cutting of their respective larval host plant (*L. tityrus*:* R. acetosa*;* L. dispar*:* R*. *hydrolapathum*;* L. helle*:* B. officinalis*). All hatchlings were kept under control conditions until allocation to treatments. Per treatment and population, 8–17 replicates were used. Again, three treatments were used: (i) control (provided with fresh cuttings under control conditions), (ii) heat (provided with fresh cuttings and exposed for 2 days to a simulated heat wave), and (iii) food stress (provided with wilted leaves to mimic the results of a period of drought for 2 days under control conditions). Control and heat conditions were identical to the first experiment (cf. Figure 2). To produce wilted leaves, leaves were cut off the plant and stored for 24 hr at 20°C and 50% r.h. in a climate cabinet without water supply. This handling resulted in levels of host plant wilting frequently experienced in the natural habitats of the species. All animals remained under control conditions before and after the treatments. The mortality rate per box was scored as percent on day 6 of larval development.

### 2.3 Statistical analyses

We analyzed mortality rates (percentage of dead individuals per box; starting with 10 individuals per box) for eggs and hatchlings using hierarchical general linear mixed models (GLMMs) with treatment and species as fixed categorical effects, and population and group (either family or random group in *L. helle*) as random categorical effects. Population was nested within species, and group was nested within species and population. Note that we used replicated populations for each species. The effect of replicate population was modeled as random effect in order to account for the variance explained by differences among populations rather than species. Group was modeled as random effect to account for the nonindependency of siblings, as a split‐brood design was used for two of the three species (see above) such that per female one group of offspring was allocated to each treatment. Separate analyses were run for eggs and hatchlings due to differences in the treatments used. Data were analyzed using Statistica 8.0 (StatSoft, Tulsa, OK, USA). Pair‐wise comparisons after GLMMs were performed employing Tukey's HSD for unequal sample sizes. Throughout the text, means are given ±1 *SE*.

## 3 RESULTS

**Figure 3:**
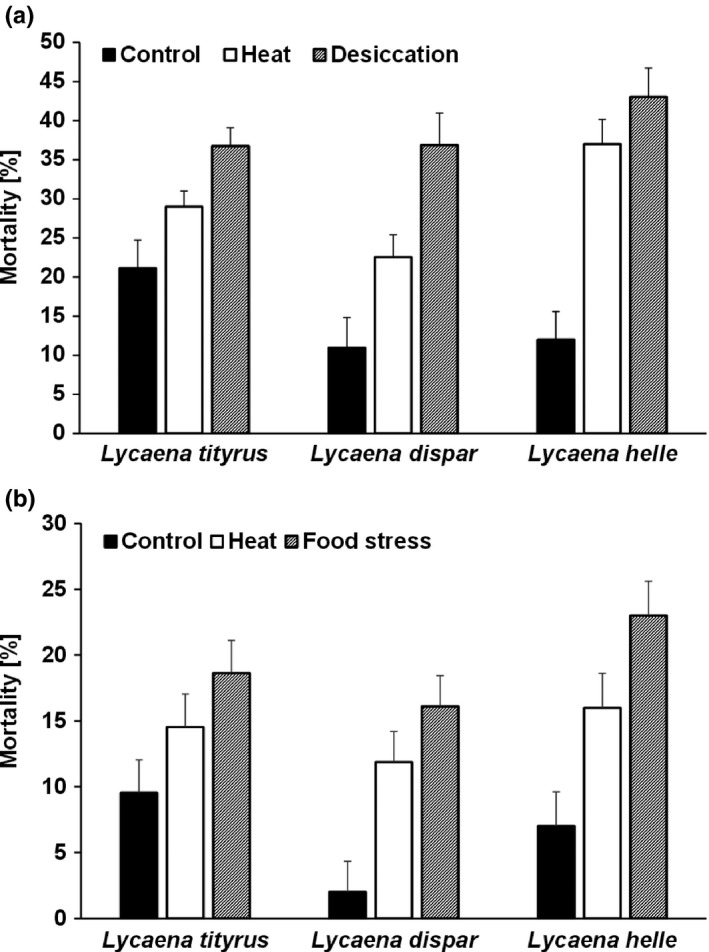
Mortality rates for eggs (a) and hatchlings (b) across three species of Copper butterflies, reflecting different vulnerabilities to climate change. Given are group means + 1 *SE*. Sample sizes range between 20 and 29 groups with 10 individuals each per treatment. For differences among treatments in experiment (a) see Figure 2. The control and heat conditions used in experiment (b) are identical to those in experiment (a), and for the food stress treatment, individuals were provided with wilted leaves for 2 days under control conditions

Average mortality rates of eggs varied significantly across treatments, being lowest under control conditions (14.2 ± 1.9%) followed by the heat (28.8 ± 1.9%) and finally the desiccation treatment (38.8 ± 1.9%; control < heat < desiccation, Tukey's HSD; Table 2a, Figure 3a). Overall, species did not differ significantly in mortality rates (*L. dispar*: 23.4 ± 1.4%; *L. tityrus*: 29.0 ± 1.9%; *L. helle*: 30.7 ± 2.0%). However, species differed in their responses to different levels of stress in egg mortality (significant species by treatment interaction). Egg mortality rate increased most strongly between control and heat/desiccation stress in *L. helle* (by 25.0 and 31.0 percentage points for the heat and desiccation treatment, respectively), but weaker in *L. dispar* (11.6 and 25.9 percentage points) and especially in *L. tityrus* (7.8 and 15.6 percentage points). Mortality rates differed significantly among populations and groups.

**Table 2:** Results of general linear mixed models (GLMMs) for the effects of treatment (control, heat, desiccation; fixed), species (fixed), population (nested within species; random), and group (nested within species and population; random) on egg (a) and hatchling (b) mortality rates in three Copper butterfly species

	MQ	*df*	*F*	*p*
(a) Eggs				
Treatment	9,933	2	67.9	**<.001**
Species	1,264	2	0.6	.562
Population (species)	1,794	3	6.4	
Group (species × pop.)	335	63	2.1	
Species × treatment	566	4	3.5	**.008**
Error	156	132		
(b) Hatchlings				
Treatment	2,878	2	21.6	**<.001**
Species	511	2	6.6	.083
Population (species)	78	3	0.5	
Group (species × pop.)	145	61	1.1	
Species × treatment	80	4	0.6	.661
Error	133	128		

MQ: mean squared sums; *df*, degree of freedom; *F*,* F*‐value; *p*,* p*‐value.

Only the results for fixed effects are presented here. Effects of population and group were significant for the egg stage only. Significant *p*‐values are given in bold.

As above, hatchling mortality differed significantly among treatments, being lowest under control conditions (6.2 ± 1.4%) followed by the heat (14.1 ± 1.4%) and finally the food stress treatment (19.2 ± 1.4%; control < heat < food stress, Tukey's HSD; Table 2b, Figure 3b). Regarding differences among species, mortality rates tended to be lower in *L. dispar* (9.9 ± 1.3%) than in *L. helle* (15.3 ± 1.5%), with *L. tityrus* showing an intermediate value (14.2 ± 1.4%). We tested whether simplification of the respective model presented in Table 2b would result in different patterns. Removing the nonsignificant factors, population and group revealed a significant species (*F*_2,136_ = 4.1, *p *= .018) and treatment effect (*F*_2,136_ = 21.1, *p *<* *.001), but once again no significant interaction (*F*_4,136_ = 0.6, *p *=* *.670).

## 4 DISCUSSION

Our experiments show that mortality rates were higher when simulating heat waves compared with control conditions. This result was expected (Andrew, Hart, Jung, Hemmings, & Terblanche, 2013; Rukke, Aak, & Edgar, 2015; Tewksbury, Huey, & Deutsch, 2008) and may be caused by, e.g., denaturation of proteins, disruption of membrane structure and function, interactions with oxygen supply, and dehydration impairing evaporative cooling (Chown & Terblanche, 2006; Klose & Robertson, 2004; Potter et al., 2009). Mortality rates of eggs further increased after exposure to low humidity, likely caused by dehydration facilitated by the low egg mass and a concomitantly high volume–surface ratio (Addo‐Bediako, Chown, & Gaston, 2001; Chown, Sørensen, & Terblanche, 2011). The level of humidity used in our treatments was very low (10%), but exposure time was quite short (2 × 2 hr). Therefore, we do think that our treatment conditions were reasonable to mimic effects of low humidity during drought periods, although it is evidently difficult to extrapolate such laboratory results to field conditions. For instance, eggs are in nature attached to host plants and may benefit from buffering microclimatic conditions (Potter et al., 2009; Smith, 1978). Furthermore, wilted as opposed to fresh leaves and the associated food stress increased hatchling mortality rates as expected. All above results are in agreement with our first hypothesis, although it is interesting to note that effects of desiccation and food stress were even more pronounced than those of heat stress. This suggests that exclusively focusing on upper thermal limits while neglecting the impact of drought periods is insufficient for predicting the fate of species and populations under changing climate (Cooper, Tharp, Jernberg, & Angilletta, 2012; Rezende, Tejedo, & Santos, 2011). Mosquito distribution, for instance, may be limited by egg desiccation resistance (Kearney, Porter, Williams, Ritchie, & Hoffmann, 2009), and fruit flies from the Australian wet tropics are known to have a low desiccation resistance and concomitant heritability, which is likely a crucial factor limiting their distribution (Hoffmann, Sørensen, & Loeschcke, 2003; Kellermann et al., 2012).

The most interesting result though is that indeed the three species investigated here differed in their responses to environmental stress, as evidenced by the significant species by treatment interaction for egg mortality, in agreement with our second hypothesis. Specifically, egg mortality increased most strongly in the arguably most vulnerable species *L. helle*, followed by *L. dispar* and finally *L. tityrus* (Figure 3a). Note that these results fit very well with our predictions based on the general ecology of the three species concerned (Ebert & Rennwald, 1991; Fischer et al., 1999; Brunzel et al., 2008; Settele et al., 2008; Lindman et al., 2015; Hampe & Jump, 2011; Table 1). While the glacial relict species *L. helle* is associated with cool and moist habitats (Fischer et al., 1999) and should therefore suffer from both, heat and desiccation (Habel et al. 2011), *L. dispar* is associated with moist but (at least partly) warm habitats (Lindman et al., 2015), such that the species should in first place suffer from desiccation rather than heat stress. Concomitantly, *L. dispar* responded less strongly to heat stress than *L. helle* but (nearly) as strongly to desiccation, while *L. tityrus*, being a habitat generalist with respect to moisture and temperature (Brunzel et al., 2008; Ebert & Rennwald, 1991), showed moderate responses only to both stressors. Note that the above conclusions rest mainly on comparisons relative to controls. This might be problematic given that *L. tityrus* showed the highest baseline mortality, which may drive the significant interaction for egg mortality (Figure 3). However, this pattern for *L. tityrus* is unusual as egg hatching success in this species is typically around 90% or higher under control conditions as found in both other species (K. Fischer, personal observation). The most likely explanation for the reduced hatching success in *L. tityrus* under control conditions seems random variation in female quality, for instance, caused by differences in female age (i.e., we assume that field‐caught *L. tityrus* females were on average a bit older than in both other species). We exclusively used field‐caught females here, as these species do not mate in captivity. To account for such effects, we are convinced that the most straightforward way to interpret our data is comparisons relative to control levels. Anyway, note in addition that *L. helle* showed the highest mortality rates under stress also in absolute terms.

In contrast to the above results on egg mortality, the respective interaction between species and treatment was not significant for hatchling mortality, although the pattern obtained was very similar (see Figure 2). These results may suggest that vulnerability to stress decreases during development, which is in line with an earlier study on the same species as investigated here, in which we found that differences in vulnerability to climate change are unlikely to be caused by differential responses to thermal and desiccation stress during (later) larval and pupal development (Klockmann, Karajoli, et al., 2016) or in the adult stage (Klockmann, Wallmeyer, et al., 2017). Thus, in the species considered here, the early developmental stage, especially the egg stage, seems to be the most critical life stage determining vulnerability to climate change.

The significant group (family) and population effects in the first experiment indicate that, besides the differences among species, heat and desiccation resistance may additionally differ among populations and families. This may again be explained by random effects including variation in the quality or condition of field‐caught females (see above). Alternatively, such variation may suggest a heritable component that can be exploited by natural selection. However, heritability in such traits is typically very low, such that evolutionary rescue appears to be unlikely given the pace of current climate change (Blackburn, van Heerwaarden, Kellermann, & Sgrò, 2014; Hoffmann, Shirriffs, & Scott, 2005; Kellermann et al., 2012).

An increased frequency of extreme weather events such as heat waves and periods of drought are important consequences of ongoing climate change (Battisti & Naylor, 2009; Coumou & Rahmstorf, 2012). Our results indicate that this may have important consequences for extant biodiversity, as simulated heat waves and drought stress generally increased mortality rates during early development, as would be expected. Our data also stress the importance of considering detrimental effects of drought when trying to forecast species responses. Importantly, we found that closely related species, arguably differing in their vulnerability to climate change, seem to differ in their responses to environmental stress. However, such variation was restricted to early developmental stages, while different levels of stress seem to have little effect on fitness during further development (i.e., in older larvae, pupae, and adults; Potter et al., 2011; Klockmann, Günter, et al., 2017; Klockmann, Karajoli, et al., 2016; Klockmann, Schröder, et al., 2016; Klockmann, Wallmeyer, et al., 2017). We suggest that, in the three species investigated here, stress tolerance during early development might be a major determinant of vulnerability to climate change and may explain recent population declines in *L. helle* along with habitat deterioration (Bauerfeind, Theisen, & Fischer, 2008; Fischer et al., 1999). These findings, if similar patterns were found in a larger array of species, may have important implications for enhancing our abilities to predict the fate of particular species and populations under ongoing climate change. For instance, it might be worth comparing models that do or do not consider differences in thermal tolerance across life stages, or models could be improved by integrating results from the arguably most sensitive stage. Recent studies on a tropical butterfly also indicated that the egg stage comprises the most vulnerable developmental stage, that body mass may be a crucial constraint on stress tolerance, and that stress experienced early in life could affect later life stages (Klockmann, Kleinschmidt, & Fischer, 2017; Klockmann, Günter, et al., 2017). Further progress regarding specific traits underlying vulnerability to climate change will likely be achieved by collating more data on stress tolerance throughout development from a broader range of taxa.

## DATA ACCESSIBILITY

After acceptance, the full dataset will be archived at external databases (e.g., DRYAD).

## CONFLICT OF INTEREST

None declared.

## AUTHOR CONTRIBUTIONS

MK and KF conceived of the study and designed methodology, and MK collected the data. Both authors wrote the manuscript and gave final approval for publication.

## References

[ece33588-bib-0001] Addo‐Bediako, A. , Chown, S. L. , & Gaston, K. J. (2001). Revisiting water loss in insects: A large scale view. Journal of Insect Physiology, 47, 1377–1388. https://doi.org/10.1016/S0022-1910(01)00128-7 12770144 10.1016/s0022-1910(01)00128-7

[ece33588-bib-0002] Anderson, A. R. , Collinge, J. E. , Hoffmann, A. A. , Kellett, M. , & McKechnie, S. W. (2003). Thermal tolerance trade‐offs associated with the right arm of chromosome 3 and marked by the hsr‐omega gene in *Drosophila melanogaster* . Heredity, 90, 195–202. https://doi.org/10.1038/sj.hdy.6800220 12634827 10.1038/sj.hdy.6800220

[ece33588-bib-0003] Andrew, N. R. , Hart, R. A. , Jung, M.‐P. , Hemmings, Z. , & Terblanche, J. S. (2013). Can temperate insects take the heat? A case study of the physiological and behavioural responses in a common ant, *Iridomyrmex purpureus* (Formicidae), with potential climate change. Journal of Insect Physiology, 59, 870–880. https://doi.org/10.1016/j.jinsphys.2013.06.003 23806604 10.1016/j.jinsphys.2013.06.003

[ece33588-bib-0004] Battisti, D. S. , & Naylor, R. L. (2009). Historical warnings of future food insecurity with unprecedented seasonal heat. Science, 323, 240–244. https://doi.org/10.1126/science.1164363 19131626 10.1126/science.1164363

[ece33588-bib-0005] Bauerfeind, S. S. , Theisen, A. , & Fischer, K. (2008). Patch occupancy in the endangered butterfly *Lycaena helle* in a fragmented landscape: Effects of habitat quality, patch size and isolation. Journal of Insect Conservation, 13, 271–277.

[ece33588-bib-0503] Beaumont, L. J. , & Hughes, L. (2002). Potential changes in the distributions of latitudinally restricted Australian butterfly species in response to climate change. Global Change Biology, 8, 954–971. https://doi.org/10.1046/j.1365-2486.2002.00490.x

[ece33588-bib-0006] Binot‐Hafke, M. , Balzer, S. , Becker, N. , Gruttke, H. , Haupt, H. , Hofbauer, N. , … Strauch, M. (2011). Rote liste gefährdeter tiere, pflanzen und pilze Deutschlands. Volume 3: Wirbellose tiere (Part 1). Bonn, Germany: Bundesamt für Naturschutz.

[ece33588-bib-0007] Blackburn, S. , van Heerwaarden, B. , Kellermann, V. , & Sgrò, C. M. (2014). Evolutionary capacity of upper thermal limits: Beyond single trait assessments. Journal of Experimental Biology, 217, 1918–1924. https://doi.org/10.1242/jeb.099184 24625644 10.1242/jeb.099184

[ece33588-bib-0008] Bowler, K. , & Terblanche, J. S. (2008). Insect thermal tolerance: What is the role of ontogeny, ageing and senescence? Biological Reviews, 83, 339–355. https://doi.org/10.1111/brv.2008.83.issue-3 18979595 10.1111/j.1469-185x.2008.00046.x

[ece33588-bib-0009] Brook, B. W. , Akçakaya, H. R. , Keith, D. A. , Mace, G. M. , Pearson, R. G. , & Araújo, M. B. (2009). Integrating bioclimate with population models to improve forecasts of species extinctions under climate change. Biology letters, 5, 723–725. https://doi.org/10.1098/rsbl.2009.0480 19625300 10.1098/rsbl.2009.0480 PMC2828003

[ece33588-bib-0010] Brunzel, S. , Bussmann, M. , & Obergruber, H. (2008). Deutliche veränderungen von tagfalterzönosen als folge von ausbreitungsprozessen. Erste ergebnisse eines monitorings über 17 jahre. Natur und Landschaft, 83, 280–287.

[ece33588-bib-0011] Chown, S. , Hoffmann, A. , Kristensen, T. , Angilletta, M. , Stenseth, N. , & Pertoldi, C. (2010). Adapting to climate change: A perspective from evolutionary physiology. Climate Research, 43, 3–15. https://doi.org/10.3354/cr00879

[ece33588-bib-0012] Chown, S. L. , Sørensen, J. G. , & Terblanche, J. S. (2011). Water loss in insects: An environmental change perspective. Journal of Insect Physiology, 57, 1070–1084. https://doi.org/10.1016/j.jinsphys.2011.05.004 21640726 10.1016/j.jinsphys.2011.05.004

[ece33588-bib-0013] Chown, S. L. , & Terblanche, J. S. (2006). Physiological diversity in insects: Ecological and evolutionary contexts. Advances in Insect Physiology, 33, 50–152. https://doi.org/10.1016/S0065-2806(06)33002-0 19212462 10.1016/S0065-2806(06)33002-0 PMC2638997

[ece33588-bib-0014] Clusella‐Trullas, S. , Blackburn, T. M. , & Chown, S. L. (2011). Climatic predictors of temperature performance curve parameters in ectotherms imply complex responses to climate change. American Naturalist, 177, 738–751. https://doi.org/10.1086/660021 10.1086/660021 21597251

[ece33588-bib-0015] Cooper, B. S. , Tharp, J. M. , Jernberg, I. I. , & Angilletta, M. J. (2012). Developmental plasticity of thermal tolerances in temperate and subtropical populations of *Drosophila melanogaster* . Journal of Thermal Biology, 37, 211–216. https://doi.org/10.1016/j.jtherbio.2012.01.001

[ece33588-bib-0016] Coumou, D. , & Rahmstorf, S. (2012). A decade of weather extremes. Nature Climate Change, 2, 1–6.

[ece33588-bib-0017] Deutsch, C. A. , Tewksbury, J. J. , Huey, R. B. , Sheldon, K. S. , Ghalambor, C. K. , Haak, D. C. , & Martin, P. R. (2008). Impacts of climate warming on terrestrial ectotherms across latitude. Proceedings of the National Academy of Sciences of the United States of America, 105, 6668–6672. https://doi.org/10.1073/pnas.0709472105 18458348 10.1073/pnas.0709472105 PMC2373333

[ece33588-bib-0019] Ebert, G. , & Rennwald, E. (1991). Die schmetterlinge baden württembergs, 2nd ed Stuttgart, Germany: Ulmer.

[ece33588-bib-0020] Fischer, K. , Beinlich, B. , & Plachter, H. (1999). Population structure, mobility and habitat preferences of the Violet Copper *Lycaena helle* (Lepidoptera: Lycaenidae) in Western Germany: Implications for conservation. Journal of Insect Conservation, 3, 43–52. https://doi.org/10.1023/A:1009630506216

[ece33588-bib-0021] Gomez, F. H. , Sambucetti, P. D. , Loeschcke, V. , & Norry, F. M. (2015). Patterns of variation in desiccation resistance in a set of recombinant inbred lines in *Drosophila melanogaster* . Physiological Entomology, 40, 205–211. https://doi.org/10.1111/phen.2015.40.issue-3

[ece33588-bib-0501] Habel, J. C. , Rödder, D. , Schmitt, T. , & Néve, G. (2011). Global warming will affect the genetic diversity and uniqueness of Lycaena helle populations. Global Change Biology, 17, 194–205. https://doi.org/10.1111/j.1365-2486.2010.02233.x

[ece33588-bib-0022] Hampe, A. , & Jump, A. S. (2011). Climate relicts: Past, present, future. Annual Review of Ecology, Evolution, and Systematics, 42, 313–333. https://doi.org/10.1146/annurev-ecolsys-102710-145015

[ece33588-bib-0023] Hansen, J. , Sato, M. , & Ruedy, R. (2012). Perception of climate change. Proceedings of the National Academy of Sciences of the United States of America, 109, E2415–E2423. https://doi.org/10.1073/pnas.1205276109 22869707 10.1073/pnas.1205276109 PMC3443154

[ece33588-bib-0502] Hoffmann, A. A. , & Sgrò, C. M. (2011). Climate change and evolutionary adaptation. Nature, 470, 479–485. https://doi.org/10.1038/nature09670 21350480 10.1038/nature09670

[ece33588-bib-0024] Hoffmann, A. A. , Chown, S. L. , & Clusella‐Trullas, S. (2013). Upper thermal limits in terrestrial ectotherms: How constrained are they? Functional Ecology, 27, 934–949. https://doi.org/10.1111/j.1365-2435.2012.02036.x

[ece33588-bib-0025] Hoffmann, A. A. , Shirriffs, J. , & Scott, M. (2005). Relative importance of plastic vs genetic factors in adaptive differentiation: Geographical variation for stress resistance in *Drosophila melanogaster* from eastern Australia. Functional Ecology, 19, 222–227. https://doi.org/10.1111/fec.2005.19.issue-2

[ece33588-bib-0026] Hoffmann, A. A. , Sørensen, J. G. , & Loeschcke, V. (2003). Adaptation of *Drosophila* to temperature extremes: Bringing together quantitative and molecular approaches. Journal of Thermal Biology, 28, 175–216. https://doi.org/10.1016/S0306-4565(02)00057-8

[ece33588-bib-0027] Kearney, M. , Porter, W. P. , Williams, C. , Ritchie, S. , & Hoffmann, A. A. (2009). Integrating biophysical models and evolutionary theory to predict climatic impacts on species’ ranges: The dengue mosquito *Aedes aegypti* in Australia. Functional Ecology, 23, 528–538. https://doi.org/10.1111/fec.2009.23.issue-3

[ece33588-bib-0028] Kellermann, V. , Overgaard, J. , Hoffmann, A. A. , Flojgaard, C. , Svenning, J.‐C. , & Loeschcke, V. (2012). Upper thermal limits of *Drosophila* are linked to species distributions and strongly constrained phylogenetically. Proceedings of the National Academy of Sciences of the United States of America, 109, 16228–16233. https://doi.org/10.1073/pnas.1207553109 22988106 10.1073/pnas.1207553109 PMC3479592

[ece33588-bib-0029] Kingsolver, J. G. (2009). The well‐temperatured biologist. American Naturalist, 174, 755–768. https://doi.org/10.1086/648310 10.1086/648310 19857158

[ece33588-bib-0030] Kingsolver, J. G. , Woods, A. H. , Buckley, L. B. , Potter, K. A. , MacLean, H. J. , & Higgins, J. K. (2011). Complex life cycles and the responses of insects to climate change. Integrative and Comparative Biology, 51, 719–732. https://doi.org/10.1093/icb/icr015 21724617 10.1093/icb/icr015

[ece33588-bib-0031] Klockmann, M. , Günter, F. , & Fischer, K. (2017). Heat resistance throughout ontogeny: Body size constrains thermal tolerance. Global Change Biology, 23, 686–696. https://doi.org/10.1111/gcb.13407 27371939 10.1111/gcb.13407

[ece33588-bib-0032] Klockmann, M. , Karajoli, F. , Reimer, S. , Kuczyk, J. , & Fischer, K. (2016). Fitness implications of simulated climate change in three species of Copper butterflies (Lepidoptera: Lycaenidae). Biological Journal of the Linnean Society, 120, 125–143. https://doi.org/10.1111/bij.12846

[ece33588-bib-0033] Klockmann, M. , Kleinschmidt, F. , & Fischer, K. (2017). Carried over: Heat stress in the egg stage reduces subsequent performance in a butterfly. PLoS ONE, 12, e0180968 https://doi.org/10.1371/journal.pone.0180968 28708887 10.1371/journal.pone.0180968 PMC5510857

[ece33588-bib-0034] Klockmann, M. , Schröder, U. , Karajoli, F. , & Fischer, K. (2016). Simulating effects of climate change under direct and diapause development in a butterfly. Entomologia Experimentalis et Applicata, 158, 60–68. https://doi.org/10.1111/eea.12380

[ece33588-bib-0035] Klockmann, M. , Wallmeyer, L. , & Fischer, K. (2017). Variation in adult stress resistance does not explain vulnerability to climate change in Copper butterflies. Insect Science, accepted https://doi.org/10.1111/1744-7917.12456 10.1111/1744-7917.12456 28294575

[ece33588-bib-0036] Klose, M. K. , & Robertson, R. M. (2004). Stress‐induced thermoprotection of neuromuscular transmission. Integrative and Comparative Biology, 44, 14–20. https://doi.org/10.1093/icb/44.1.14 21680481 10.1093/icb/44.1.14

[ece33588-bib-0037] Limberg, J. , & Fischer, K. (2014). Blauschillernder Feuerfalter *Lycaena helle* (D & S, 1975) In Kerth G (Ed.), Anpassungskapazität ausgewählter Arten im Hinblick auf Änderungen durch den Klimawandel, (pp. 204–212). Bonn: BfN. Naturschutz und Biologische Vielfalt.

[ece33588-bib-0038] Lindman, L. , Remm, J. , Saksing, K. , Sõber, V. , Õunap, E. , & Tammaru, T. (2015). *Lycaena dispar* on its northern distribution limit: An expansive generalist. Insect Conservation and Diversity, 8, 3–16. https://doi.org/10.1111/icad.2015.8.issue-1

[ece33588-bib-0040] Martin, Y. , Van Dyck, H. , Dendoncker, N. , & Titeux, N. (2013). Testing instead of assuming the importance of land use change scenarios to model species distributions under climate change. Global Ecology and Biogeography, 22, 1204–1216. https://doi.org/10.1111/geb.2013.22.issue-11

[ece33588-bib-0041] McKechnie, A. E. , & Wolf, B. O. (2010). Climate change increases the likelihood of catastrophic avian mortality events during extreme heat waves. Biology Letters, 6, 253–256. https://doi.org/10.1098/rsbl.2009.0702 19793742 10.1098/rsbl.2009.0702 PMC2865035

[ece33588-bib-0042] Meehl, G. A. , et al. (2007). Global climate projections In Solomon S (Ed.), Climate change 2007: The physical science basis. Contribution of working group i to the fourth assessment report of the intergovernmental panel on climate change (pp. 747–845). Cambridge, UK: Cambridge University Press.

[ece33588-bib-0043] Parmesan, C. , & Yohe, G. (2003). A globally coherent fingerprint of climate change impacts across natural systems. Nature, 421, 37–42. https://doi.org/10.1038/nature01286 12511946 10.1038/nature01286

[ece33588-bib-0044] Pichrtová, M. , Kulichová, J. , Holzinger, A. (2014). Nitrogen limitation and slow drying induce desiccation tolerance in conjugating green algae (Zygnematophyceae, Streptophyta) from polar habitats (ed J‐S Zhang). PLoS ONE, 9, e113137 https://doi.org/10.1371/journal.pone.0113137 25398135 10.1371/journal.pone.0113137 PMC4232603

[ece33588-bib-0045] Pimm, S. L. , Jenkins, CN , Abell, R , Brooks, TM , Gittleman, JL , Joppa, LN , … Sexton, JO (2014). The biodiversity of species and their rates of extinction, distribution, and protection. Science, 344, 1246752 https://doi.org/10.1126/science.1246752 24876501 10.1126/science.1246752

[ece33588-bib-0046] Potter, K. A. , Davidowitz, G. , & Arthur Woods, H. (2011). Cross‐stage consequences of egg temperature in the insect Manduca sexta. Functional Ecology, 25, 548–556. https://doi.org/10.1111/fec.2011.25.issue-3

[ece33588-bib-0047] Potter, K. , Davidowitz, G. , & Woods, H. A. (2009). Insect eggs protected from high temperatures by limited homeothermy of plant leaves. Journal of Experimental Biology, 212, 3448–3454. https://doi.org/10.1242/jeb.033365 19837886 10.1242/jeb.033365

[ece33588-bib-0048] Radchuk, V. , Turlure, C. , & Schtickzelle, N. (2013). Each life stage matters: The importance of assessing the response to climate change over the complete life cycle in butterflies. Journal of Animal Ecology, 82, 275–285. https://doi.org/10.1111/j.1365-2656.2012.02029.x 22924795 10.1111/j.1365-2656.2012.02029.x

[ece33588-bib-0049] Rezende, E. L. , Tejedo, M. , & Santos, M. (2011). Estimating the adaptive potential of critical thermal limits: Methodological problems and evolutionary implications. Functional Ecology, 25, 111–121. https://doi.org/10.1111/j.1365-2435.2010.01778.x

[ece33588-bib-0504] Rosset, V. , & Oertli, B. (2011). Freshwater biodiversity under climate warming pressure: identifying the winners and losers in temperate standing waterbodies. Biological Conservation, 144, 2311–2319. https://doi.org/10.1016/j.biocon.2011.06.009

[ece33588-bib-0050] Rukke, B. A. , Aak, A. , & Edgar, K. S. (2015). Mortality, temporary sterilization, and maternal effects of sublethal heat in bed bugs. PLoS ONE, 10, e0127555 https://doi.org/10.1371/journal.pone.0127555 25996999 10.1371/journal.pone.0127555 PMC4440821

[ece33588-bib-0051] Settele, J. , et al. (2008). Climatic risk atlas of European butterflies. Sofia‐Moscow, Bulgaria: Pensoft.

[ece33588-bib-0053] Smith, W. K. (1978). Temperatures of desert plants: Another perspective on the adaptability of leaf size. Science, 201, 614–616. https://doi.org/10.1126/science.201.4356.614 17794122 10.1126/science.201.4356.614

[ece33588-bib-0054] Sunday, J. M. , Bates, A. E. , & Dulvy, N. K. (2012). Thermal tolerance and the global redistribution of animals. Nature Climate Change, 2, 686–690. https://doi.org/10.1038/nclimate1539

[ece33588-bib-0055] Tejeda, M. T. , Arredondo, J , Liedo, P , Pérez‐Staples, D , Ramos‐Morales, P , Díaz‐Fleischer, F (2016). Reasons for success: Rapid evolution for desiccation resistance and life‐history changes in the polyphagous fly *Anastrepha ludens* . Evolution, 11, 2583–2594. https://doi.org/10.1111/evo.13070 10.1111/evo.13070 27641541

[ece33588-bib-0056] Tewksbury, J. J. , Huey, R. B. , & Deutsch, C. A. (2008). Putting the heat on tropical animals. Science, 320, 1296–1297. https://doi.org/10.1126/science.1159328 18535231 10.1126/science.1159328

[ece33588-bib-0057] Thomas, C. D. (2010). Climate, climate change and range boundaries. Diversity and Distributions, 16, 488–495. https://doi.org/10.1111/j.1472-4642.2010.00642.x

[ece33588-bib-0058] Thomas, C. D. , Cameron, A , Green, R.E. , Bakkenes, M , Beaumont, L.J. , Collingham, Y.C. , … Hughes, L (2004). Extinction risk from climate change. Nature, 427, 145–148. https://doi.org/10.1038/nature02121 14712274 10.1038/nature02121

[ece33588-bib-0059] Tolman, T. , & Lewington, R. (2008). Collins butterfly guide: The most complete field guide to the butterflies of Britain and Europe. London, UK: HarperCollins.

[ece33588-bib-0060] Van Swaay, C. A. M. , & Warren, M. S. (1999). Red data book of European butterflies (Rhopalocera), nature and environment No. 99. Strasbourg, France: Council of Europe Publishing.

[ece33588-bib-0061] Williams, S. E. , Shoo, L. P. , Isaac, J. L. , Hoffmann, A. A. , & Langham, G. (2008). Towards an integrated framework for assessing the vulnerability of species to climate change. PLoS biology, 6, 2621–2626. https://doi.org/10.1371/journal.pbio.0060325 19108608 10.1371/journal.pbio.0060325 PMC2605927

[ece33588-bib-0062] Woods, H. A. (2013). Ontogenetic changes in the body temperature of an insect herbivore. Functional Ecology, 27, 1322–1331. https://doi.org/10.1111/1365-2435.12124

[ece33588-bib-0063] Zimmermann, N. E. , Edwards, NG , Edwards, TC , Meier, ES , Thuiller, W , Guisan, A , … Pearman, PB (2009). Climatic extremes improve predictions of spatial patterns of tree species. Proceedings of the National Academy of Sciences of the United States of America, 106, 19723–19728. https://doi.org/10.1073/pnas.0901643106 19897732 10.1073/pnas.0901643106 PMC2780931

